# The palm or the plate? An assessment of dietary guideline promotion, awareness, and preferences among Saudis: cross-sectional survey

**DOI:** 10.1186/s12889-023-16435-8

**Published:** 2023-10-12

**Authors:** Aroub Alnasser

**Affiliations:** Food Science and Nutrition Department, College of Food and Agriculture Sciences, King Saud University, P.O. Box 22452, 11495 Riyadh, Saudi Arabia

**Keywords:** Dietary guidelines, The Healthy Food Palm, The Saudi Healthy Plate, Food education, Food-related policies

## Abstract

**Background:**

Dietary Guidelines for Saudis are offered and promoted in two forms: the Healthy Food Palm and the Saudi Healthy Plate. However, public awareness, recognition, and engagement with these guidelines is not well studied. Understanding the factors behind dietary guideline promotion, awareness, recognition, and use may lead to greater optimization of and engagement with the guidelines. This study aims to assess recognition of and public engagement with the My Palm and My Plate dietary guidelines as well as dietary guideline awareness, perceptions, and preferences among Saudis.

**Methods:**

To compare awareness, knowledge, and usability between the Healthy Food Palm and the Saudi Healthy Plate dietary guidelines, a cross-sectional survey-based study was conducted among Saudi adult males and females above 18 (*n* = 674) between October 2021 and January 2022. Participants were split into groups based on age and gender. *T*-test and χ2 tests were used to determine differences between groups for continuous and categorical variables, respectively.

**Results:**

Most participants were unaware of the Healthy Food Palm and the Saudi Healthy Plate guidelines, with only 11.1% and 30.3% able to recognize guideline-associated visual illustrations, respectively. However, nearly half of the participants correctly identified the number of food groups in the Healthy Food Palm and Saudi Healthy Plate. As well, more than half of the sample preferred the Saudi Healthy Plate, while one-quarter of the sample preferred neither guideline. In terms of future public health promotion, participants identified that “convenience and availability” (29.6%) and “my own health” (28.6%) were the most influential dietary choice factors with “social media campaigns” (2.4%) and the involvement of “an influencer” (3.1%) being the least influential dietary choice factors.

**Conclusions:**

Implementation of and public awareness of dietary guidelines is less than optimal, and opportunities exist for greater information dissemination and public engagement. Measurement and ongoing evaluation of public dietary guideline awareness and use and a more in-depth understanding of dietary choice influences and behaviors are important considerations for dietary guideline development and promotion.

## Introduction

Food-based dietary guidelines (FBDGs) are specific guidelines established by countries in order to provide citizens with recommendations on how to eat healthily [1]. Generally, FBDGs are composed of science-based messaging, easily translated for the general public’s benefit, along with simple infographics and resources to ensure the messages can be easily understood [2]. The framework for FBDGs has been laid out by organizations like the World Health Organization (WHO) and the Food and Agriculture Organization (FAO) [3]. These groups suggest that FBDGs should be realistic, attainable, and culturally sensitive [4].

Since the FAO and WHO provided guidelines for implementing FBDGs in the 1990s [3], many countries have created various dietary guidelines and recommendations based on the FAO and WHO FBDG framework. Indeed, the WHO and FAO suggested that FBDGs need to be culturally sensitive and developed within the context of a specific country [5].

In Saudi Arabia, the Ministry of Health first issued the Healthy Food Palm dietary guideline in 2012 [6, 7] to provide dietary guidance to Saudi population. Graphically represented as an easily recognizable palm tree, the Healthy Food Palm presents food groupings and associative servings, similar to the USDA Food Guide Pyramid [8]. Subsequently, and contrastingly, in 2020, the Saudi Food and Drug Authority (SFDA) issued the Saudi Healthy Plate dietary guideline [9]. The Saudi Healthy Plate visually depicts five food groups, similar to the Eatwell Plate UK [10]. Both Saudi dietary guidelines encourage healthy and diverse eating options. Since the inception of both the Healthy Food Palm and the Saudi Healthy Plate, however, little research has focused on or ascertained public awareness of and adherence to either guideline and their effects on improving Saudis’ diets.

In fact, a literature review of post-2012 research revealed that few studies have assessed adherence to or awareness of the Healthy Food Palm and the Saudi Healthy Food Plate guidelines. Two cross-sectional studies conducted in 2019 [11, 12] did measure adherence to the Healthy Food Palm among Saudis. Results from Halawani et al. [11] showed that Saudis “do not adhere” to the Healthy Food Palm, and in general, the dietary intake of normal BMI, undergraduate, and single participants were lower than the recommended range comparatively. Another study [12] measured adherence to the Healthy Food Palm among Saudi males with and without cardiovascular diseases (CVD). They found that the non-CVD individuals consumed more fruit, olive oil, and non-alcoholic beer compared to individuals with CVD. In terms of guideline awareness, a survey study [13] that explored the views of Saudi male teachers on teaching nutrition in public high schools discovered that a majority of participants (73%) were aware of the American Food Pyramid, yet only 36% were aware of the Saudi Healthy Food Palm guideline.

A recent literature survey [14] investigated the awareness or use of dietary guidelines among Saudi mothers. Interestingly, the study assessed participant knowledge of American dietary guidelines, not Saudi dietary guidelines. To the best of our knowledge, there has been a dearth of studies assessing what differing effects, if any, the Healthy Plate versus the Healthy Palm has had on Saudi dietary habits. Given this, the aim of this survey study is to assess Saudi awareness, perceptions, and preferences in terms of the Healthy Palm and the Saudi Healthy Plate dietary guidelines.

## Methods

### Study design and participants

A cross-sectional study with a convenient sampling method was conducted from October 2021 to January 2022. Using Google Forms, an online questionnaire was created, and the survey questionnaire was promoted through three popular social media apps (Twitter, Telegram, and WhatsApp) which was appropriate for selecting the sample in this research. This method was suitable considering the timing of the study's data collection, which coincided with the COVID-19 pandemic that hindered direct contact among individuals in the community. Additionally, this method may provide a significant opportunity to represent the population. The researcher consciously distributed the survey via diverse online social media platforms. According to the Communications and Information Technology Commission (CITC) of the government of Saudi Arabia, 97.8% of the total population are on one or more social media platforms [15].

After the initial stage of convenience sampling, snowball sampling was used to extend and optimize the survey’s outreach and efficacy.The researcher encouraged participants to share links with their social networks. Though nevertheless biased against those who are not in these majorities, the survey distribution method was purposefully employed for maximum exposure and outreach among Saudis.

The inclusion criteria included male and female Saudi adults (older than18 years of age). Data was collected in a safe and cost and time-effective manner. The timeline of the survey during COVID-19 restrictions guided the survey process toward online, contactless recruitment, survey administration, and data collection. As well as the popularity of apps such as WhatsApp, Twitter, and Telegram among Saudis [16] and the potential of successful snowball sampling after initial social media recruitment shaped the study’s design and recruitment.

#### Ethics approval

The study was conducted in accordance with the Declaration of Helsinki and was approved by the ethical committee of King Saud University with reference number (KSU-HE-21–674). Once participants clicked on the study’s hyperlink, the study’s title and aim appeared. In order to continue, participants had to review the study’s aim and agree to continue. If the participant did not agree (did not provide informed consent), they were not granted access to the study.

### Questionnaire development

An online self-administered survey was developed following the Checklist for Reporting Results of Internet E-Surveys (CHERRIES) guidelines [17] and Strengthening the Reporting of Observational Studies in Epidemiology (STROBE) guidelines [18]. Healthy Food Palm and the Saudi Healthy Plate guidelines. The survey included official graphics from the guidelines themselves. Using the general principles of good survey design [19], the questionnaire was created and divided into five sections. The first section assessed the demographic breakdown of survey participants (age, gender, weight, height, marital status, and education level). BMI was calculated using participants’ self-reported height and weight.

In the second section of the survey, Healthy Food Palm and Saudi Healthy Plate dietary guideline awareness and familiarity were ascertained through yes-or-no-unsure questions: Have you heard of the dietary guidelines for Saudis: the Healthy Food Palm? and Have you seen the Healthy Food Palm? The next question showed the official images for both guidelines to determine whether or not the participant recognized the image. These image-recognition questions were adapted from the Tagtow & Raghavan [20] study and modified in accordance with the research objectives.

Questions in the third section were partly adapted from a study by Schwartz & Vernarelli [21] and assessed knowledge and practice of the dietary guidelines and knowledge of the recommended food groups and serving sizes. The questions asked included the following: How many food groups are in the Saudi Healthy Food Palm? How many servings of cereal and bread are recommended by the Saudi Healthy Food Palm daily? How many servings of fruit are recommended by the Saudi Healthy Food Palm daily? And How many food groups are in the Saudi Healthy Plate? How many servings of the fruit and vegetable group is recommended by Saudi Healthy Plate? Based on the Saudi Healthy Plate guidelines, starchy foods should comprise how much of the food we eat?

The fourth section measured participant perception of the usability and usefulness of the two dietary guidelines. Three yes-or-no-unsure questions focused on the following statements: The Healthy Food Palm/The Saudi Healthy Plate is easy to understand; I follow The Healthy Food Palm/The Saudi Healthy Plate; I would recommend the Healthy Food Palm/The Saudi Healthy Plate; and The Healthy Food Palm/The Saudi Healthy Plate helps me eat better. If they answered 'yes' to the last question, participants were asked a follow up question: During the past 7 days, how many times did you eat fruit and vegetable?

In the fifth and final survey section, one question compared participants’ dietary guideline preferences, I prefer to use The Healthy Food Palm; Saudi Healthy Plate; Neither and another question asked What influences your dietary choices, including any changes in diet?

To determine the questionnaire content validity, the first draft of the survey was reviewed by a diverse panel of five knowledge-area experts and research professionals. This panel consisted of two health experts from the Ministry of Health, one from the Saudi Food and Drug Administration (SFDA), one academic expert with surveys who had conducted several surveys, and one academic statistician. To ensure face validity, the revised questionnaire was pretested with 25 subjects and refined accordingly.

### Statistical analysis

The estimated sample size was derived from the online Raosoft sample size calculator [22], taking into account the current population of 12.6 million Saudi adults aged ≥ 18 years old [23]. The sample size was set at 385 participants, based on a response rate of 50% [24–26], a confidence interval of 99% and a margin of error of 5%. Data obtained were analyzed using the Statistical Package of Social Sciences (SPSS) system (Version 22.0). Descriptive statistics (frequency, percentages, and cross-tabulation), Chi-Square test, and a t-test. A significant result means that the *P*-value for the hypothesis tests is less than 0.05. The confidence intervals (CI) were reported as 95%. The sample of this study was divided into three age groups based on cumulative percentage; 18 to 26 years (*n* = 204); 27 to 37 years (*n* = 228); and ≥ 38 years (*n* = 242).

## Results

### Characteristics

The survey had 683 respondents. Five people (0.7%) declined to participate, and four responses (0.6%) were removed due to incomplete/missing information such as weight; height, or inconsistent data such as age as below 18 years. A total of 674 responses were used for further analysis.

The sociodemographic characteristics of all participants stratified by gender (Female, Male) are presented in Table 1. The majority of the respondents were female 76% (515/674), while 23.6% (159/674) were male. The mean age of the respondents was 33.9 ± 11.7 years old (32.7 ± 11.9 for males vs. 34.2 ± 11.6 for females). However, there was no statistical difference between male and female participants according to the mean ± SD of age (*P*-value > 0.05).

**Table 1:** Sociodemographic characteristics by gender of the 674 Saudi adult participants in the Saudi national dietary guidelines cross sectional study

Variables	Gender		Total	*P*-Value
	**Male**	**Female**		
**Age** in years (mean ± SD)	32.7 ± 11.9	34.2 ± 11.6	33.9 ± 11.7	.16
**BMI** kg/m2 (mean ± SD)	27.1 ± 6.4	26.1 ± 6.4	26.4 ± 6.4	.09
**Education level**				
Up to high school	22 (13.8%)	71 (13.8%)	93 (13.8%)	.34
Undergraduate	94 (59.1%)	274 (53.2%)	368 (54.6)	
Graduate & postgraduate	43(27%)	170 (33%)	213 (31.6)	
**BMI**				
Underweight	10 (6.3%)	35 (6.8%)	45 (6.7%)	.49
Normal weight	59 (37.1%)	222 (43.1%)	281 (41.7%)	
Overweight	50 (31.4%)	152 (29.5%)	202 (30.0%)	
Obesity	40 (25.2%)	106 (20.6%)	146 (21.7%)	
**Total**	159 (100%)	515 (100%)	674 (100%)	

In terms of the other two demographic characteristics measured, BMI and education level, almost half of the sample 51.7% (348/674) was overweight or obese. Regarding education level, undergraduates comprised 54.6% (368/674), while graduates and postgraduates made up 31.6% (213/674). However, in regards to BMI and education level (*P*-value > 0.05 for all), there was no statistically significant difference between male and female participants.

### Awareness of the dietary guidelines

Dietary guideline awareness by age group is presented in Table 2. The majority of participants were not aware of the Healthy Food Palm and the Saudi Healthy Plate guidelines 84.4% (569/674) and 84.9% (572/674), respectively, with only 11.1% (75/674) and 30.3% (204/674) able to recognize the visual illustrations represented. A significantly greater percentage of Saudi adults in the age group of 27–37 years old were more aware and familiar with the Healthy Food Palm program as compared to other age groups (*P* = 0.033, *P* = 0.036).

**Table 2:** Saudi national dietary guideline awareness by age group of the 674 Saudi adult participants in the Saudi national dietary guidelines cross sectional study

Variables	Age group			Total	*P*-Value
	**18 to 26**	**27 to 37**	** > 38**		
**Have you heard of the dietary guidelines for Saudis: the Healthy Food Palm?**					
No	179 (87.7%)	181 (79.4%)	209 (86.4%)	569 (84.4%)	.03
Yes	25 (12.3%)	47 (20.6%)	33 (13.6%)	105 (15.6%)	
Total	204 (100%)	228 (100%)	242 (100%)	674 (100%)	
**Have you seen the Healthy Food Palm image previously?**					
No	151 (74.0%)	172 (75.4%)	196 (81.0%)	519 (77.0%)	.04
Don’t know	22 (10.8%)	11 (4.8%)	15 (6.2%)	48 (7.1%)	
Yes	31 (15.2%)	45 (19.7%)	31 (12.8%)	107 (15.9%)	
Total	204 (100%)	228 (100%)	242 (100%)	674 (100%)	
**Have you heard of the Saudi Healthy Plate?**					
No	174 (85.3%)	187 (82.0%)	211 (87.2%)	572 (84.9%)	.29
Yes	30 (14.7%)	41 (18.0%)	31 (12.8%)	102 (15.1%)	
Total	204 (100%)	228 (100%)	242 (100%)	674 (100%)	
**Have you seen the Saudi Healthy Plate image previously?**					
No	94 (46.1%)	142 (62.3%)	159 (65.7%)	395 (58.6%)	< .001
Don’t know	28 (13.7%)	21 (9.2%)	26 (10.7%)	75 (11.1%)	
Yes	82 (40.2%)	65 (28.5%)	57 (23.6%)	204 (30.3%)	
Total	124 (60.8%)	100 (43.9%)	89 (36.8%)	313 (46.4%)	

### Knowledge of the dietary guidelines

Nearly half of the participants correctly identified the number of food groups in the Healthy Food Palm 53% (357/674) and Saudi Healthy Plate 53.9% (363/674), respectively. For the questions that asked respondents to identify daily recommended portions of cereal & bread and the fruit group, 53.2% (237/674) and 32.8% (221/674), of respondents, answered ‘Do not know’ for the Saudi Healthy Food Palm or Saudi Health Plate image respectively.

The number of people correctly identifying the number of fruit and vegetable portions was low 6.28% (423/674) on the Saudi Healthy Plate image, while the starchy foods group was more frequently correctly identified 68.1% (459/674).

The study recorded statistically significant differences between the three age groups in terms of the response to the question, “How many food groups are in the Saudi Healthy Plate?” (*P* = 0.001, *P* < 0.001, *P* = 0.001, *P* = 0.001, *P* = 0.001 and *P* = 0.003, respectively) (Table 3).

**Table 3:** Saudi national dietary guideline knowledge by age group of the 674 Saudi adult participants in the Saudi national dietary guidelines cross sectional study

Variables	Age group			Total	*P*-Value
	**18 to 26**	**27 to 37**	** > 38**		
**How many food groups are in the Saudi Healthy Food Palm?**					
Don’t know	14 (6.9%)	27 (11.8%)	43 (17.8%)	84 (12.5%)	< .001
4	4 (2.0%)	4 (1.8%)	10 (4.1%)	18 (2.7%)	
5	26 (12.7%)	29 (12.7%)	30 (12.4%)	85 (12.6)	
6	42 (20.6%)	58 (25.4%)	30 (12.4%)	130 (19.3%)	
7	118 (57.8%)	110 (48.2%)	129 (53.3%)	357 (53.0%)	
Total	204 (100%)	228 (100%)	242 (100%)	674 (100%)	
**How many servings of cereal and bread are recommended by the Saudi Healthy Food Palm daily?**					
Don’t know	51 (25.0%)	89 (39.0%)	97 (40.1%)	237 (53.2%)	< 0.001
2–4	14 (6.9%)	29 (12.7%)	48 (19.8%)	91 (13.5%)	
3–5	15 (7.4%)	10 (4.4%)	8 (3.3%)	33 (4.9%)	
6–11	124 (60.8%)	100 (43.9%)	89 (36.8%)	313 (46.4%)	
Total	204 (100%)	228 (100%)	242 (100%)	674 (100%)	
**How many servings of fruit are recommended by the Saudi Healthy Food Palm daily?**					
Don’t know	48 (23.5%)	86 (37.7%)	87 (36.0%)	221 (32.8%)	< 0.001
2–4	119 (58.3%)	110 (48.2%)	102 (42.1%)	331 (49.1%)	
3–5	27 (13.2%)	28 (12.3)	35 (14.5%)	90 (13.4%)	
6–11	10 (4.9%)	4 (1.8%)	18 (7.4%)	32 (4.7%)	
Total	204 (100%)	228 (100%)	242 (100%)	674 (100%)	
**How many food groups are in the Saudi Healthy Plate?**					
Don’t know	17 (8.3%)	29 (12.7%)	29 (12.0%)	75 (11.1%)	< 0.001
4	43 (21.1%)	58 (25.4%)	96 (39.7%)	197 (29.2%)	
5	124 (60.8%)	132 (57.9%)	107 (44.2%)	363 (53.9%)	
6	14 (6.9%)	5 (2.2%)	4 (1.7%)	23 (3.4%)	
7	6 (2.9%)	4 (1.8%)	6 (2.5%)	16 (2.4%)	
Total	204 (100%)	228 (100%)	242 (100%)	674 (100%)	
**How many servings of the fruit and vegetable group are recommended by Saudi Healthy Plate?**					
Don’t know	35 (17.2)	61 (26.8%)	67 (27.7%)	163 (24.2%)	.003
3	19 (9.3%)	21 (9.2%)	22 (9.1%)	62 (9.2%)	
5	144 (70.6%)	143 (62.7%)	136 (56.2)	423 (6.28%)	
7	6 (2.9%)	3 (1.3%)	17 (7.0%)	26 (3.9%)	
Total	204 (100%)	228 (100%)	242 (100%)	674 (100%)	
**Based on the Saudi Healthy Plate guidelines, starchy foods should comprise -**					
Don’t know	36 (17.6%)	57 (25.0%)	60 (24.8%)	153 (22.7%)	.36
All of the food we eat	1 (0.5%)	1 (0.4%)	2 (0.8%)	4 (0.6%)	
1/3 of the food we eat	150 (73.5%)	154 (67.5%)	155 (64.0%)	459 (68.1%)	
1/2 of the food we eat	17 (8.3%)	16 (7.0%)	25 (10.3%)	58 (8.6%)	
Total	204 (100%)	228 (100%)	242 (100%)	674 (100%)	

### Usability or perception of the dietary guidelines

The perceived usability of the dietary guidelines of the study population is vetted in Table 4. It can be seen that 53.6% (36/674) of respondents agreed that the Healthy Food Palm was easy to understand with 31.6% (213/674) indicating uncertainty by responding “Unsure.”

**Table 4:** Saudi national dietary guideline usability or perception of the dietary guidelines awareness by age group of the 674 Saudi adult participants in the Saudi national dietary guidelines cross sectional study

Variables	Age group			Total	*P*-Value
	**18 to 26**	**27 to 37**	** > 38**		
**The Healthy Food Palm is easy to understand**					
No	35 (17.2%)	36 (15.8%)	29 (12.0%)	100 (14.8%)	.11
Don’t know	51 (25.0%)	76 (33.3%)	86 (35.5%)	213 (31.6%)	
Yes	118 (57.8%)	116 (50.9%)	127 (52.5%)	361 (53.6%)	
Total	204 (100%)	228 (100%)	242 (100%)	674 (100%)	
**I follow the Healthy Food Palm**					
Don’t know	24 (11.8%)	89 (39.0%)	97 (40.1%)	237 (35.2%)	.003
No	124 (60.8%)	29 (12.7%)	48 (19.8%)	91 (13.5%)	
Sometimes	53 (26.0%)	10 (4.4%)	8 (3.3%)	33 (4.9%)	
Yes	3 (1.5%)	100 (43.9%)	89 (36.8%)	313 (46.4%)	
Total	204 (100%)	228 (100%)	242 (100%)	674 (100%)	
**The Healthy Food Palm helps me eat better**					
No	41 (20.1%)	46 (20.2%)	27 (11.2%)	114 (16.9%)	.007
Don’t know	69 (33.8%)	97 (42.5%)	114 (47.1)	280 (41.5%)	
Yes	94 (46.1%)	85 (37.3%)	101 (41.7%)	280 (41.5%)	
Total	204 (100%)	228 (100%)	242 (100%)	674 (100%)	
**I would recommend the Healthy Food Palm**					
No	32 (15.7%)	27 (11.8%)	17 (7.0%)	76 (11.3%)	< 0.001
Don’t know	51 (25.0%)	95 (40.5%)	98 (40.5%)	244 (36.2%)	
Yes	121 (59.3%)	106 (46.5%)	127 (52.5%)	354 (52.5%)	
Total	204 (100%)	228 (100%)	242 (100%)	674 (100%)	
**Is the Saudi Healthy Plate easy to understand?**					
No	23 (11.3%)	18 (7.9%)	24 (9.9%)	65 (9.6%)	.006
Don’t know	32 (15.7%)	66 (28.9%)	71 (29.3%)	169 (25.1%)	
Yes	149 (73.0%)	144 (63.2%)	147 (60.7%)	440 (65.3%)	
Total	204 (100%)	228 (100%)	242 (100%)	674 (100%)	
**Are you following the Saudi Healthy Plate guidelines?**					
Don’t know	22 (10.8%)	37 (16.2%)	51 (21.1%)	110 (16.3%)	.002
No	102 (50.0%)	96 (42.1%)	73 (30.2%)	271 (40.2%)	
Sometimes	62 (30.4%)	71 (31.1)	90 (37.2%)	223 (33.1%)	
Yes	18 (8.8%)	24 (10.5%)	28 (11.6%)	70 (10.4%)	
Total	204 (100%)	228 (100%)	242 (100%)	674 (100%)	
**Do you think the Saudi Healthy Plate guidelines are helpful?**					
No	13 (6.4%)	6 (2.6%)	6 (2.5%)	25 (3.7%)	< 0.001
Don’t know	26 (12.7%)	64 (30.6%)	74 (30.6%)	164 (24.3%)	
Yes	165 (80.9%)	158 (69.3%)	162 (66.9%)	485 (72.0%)	
Total	204 (100%)	228 (100%)	242 (100%)	674 (100%)	
**Would you recommend the Saudi Healthy Plate?**					
No	21 (10.3%)	15 (6.6%)	7 (2.9%)	43 (6.4%)	< 0.001
Don’t know	29 (14.2%)	74 (32.5%)	84 (34.7%)	187 (27.7%)	
Yes	154 (75.5%)	139 (61.0%)	151 (62.4%)	444 (65.9%)	
Total	204 (100%)	228 (100%)	242 (100%)	674 (100%)	

**Fig. 1:**
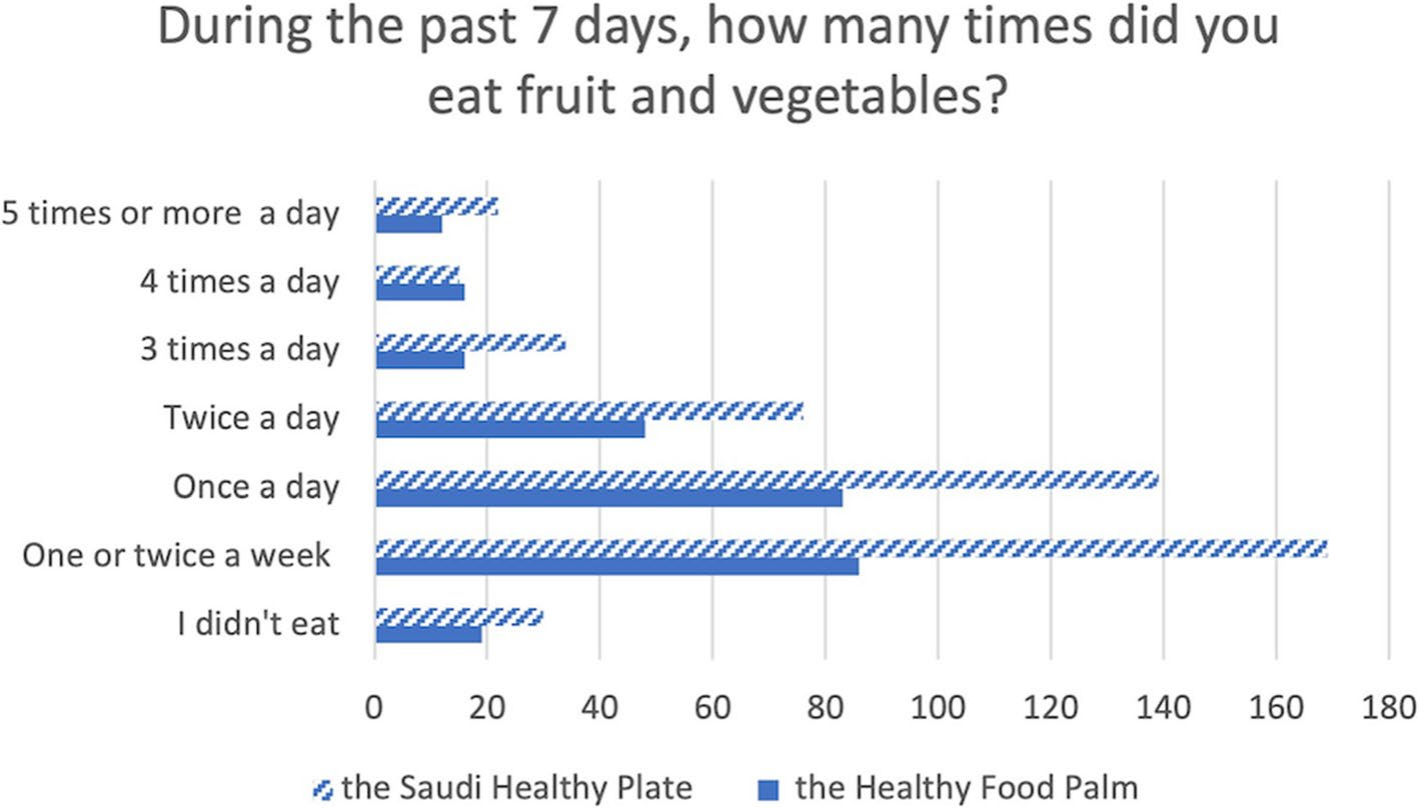
Daily self-reported fruit and vegetable intake over a seven-day cycle as presented in a bar graph

In terms of implementing dietary guidelines, 53.2% (237/674) indicated they “do not know” if they were following the Healthy Food Palm. Though 41.5% (280/674) reported that the Healthy Food Palm helps them eat better, only 4.3% (12/280) eat 5 portions a day of fruit and vegetable (Fig. 1). A small group (11.3%) would not recommend the Healthy Food Palm to others.

In comparison, 65.3% (440/674) of respondents agreed that the Saudi Healthy Plate was easy to understand, and the majority 65.9% (444/674) would recommend it to others. However, only 10.4% (70/674) pointed out they are following the Saudi Healthy Plate. While 72.0% (485/674) of participants considered the guideline helpful, only 4.5% (22/485) indicated that they eat 5 portions a day of fruit and vegetable (Fig. 1).

### Comparison of dietary guideline preferences for Saudis

A preferential comparison between the Healthy Food Palm and Saudi Healthy Plate is laid out in Table 5. In terms of exploring the preferred dietary guidelines, a slight majority 53.6% (361/674) preferred to use the Saudi Healthy Plate, while 25.1% (169/674) did not prefer to use any of the guidelines.

**Table 5:** Comparative Saudi national dietary guideline usage preference by age group of the 674 Saudi adult participants in the Saudi national dietary guidelines cross sectional study

Variables	Age group			Total	*P*-Value
	**18 to 26**	**27 to 37**	** > 38**		
I prefer to use					
Neither	40 (19.6%)	59 (25.9%)	70 (28.9%)	169 (25.1%)	.18
The Saudi Healthy Plate	122 (59.8%)	119 (52.2%)	120 (49.6%)	361 (53.6%)	
The Healthy Food Palm	42 (20.6%)	50 (21.9%)	52 (21.5%)	144 (21.4%)	

**Fig. 2:**
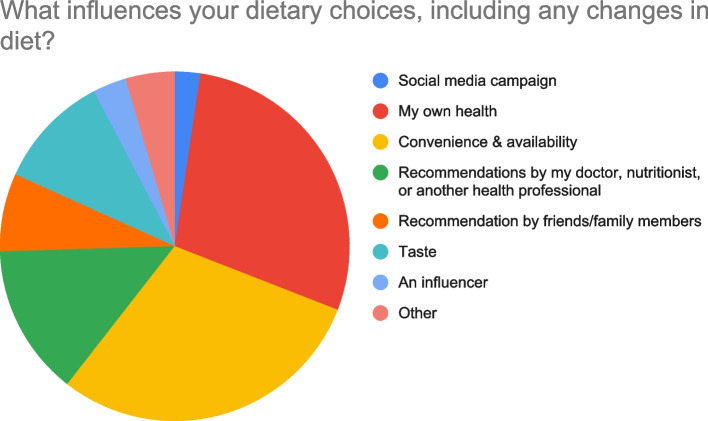
Factors influence dietary choice and consumption as presented in a pie chart

In terms of public health dietary guideline promotion and dietary choice influences, participants identified that “convenience and availability” (29.6%) and “my own health” (28.6%) were the most influential dietary choice factors with “social media campaigns” (2.4%) and the involvement of “an influencer” (3.1%) being the least influential dietary choice factors (Fig. 2).

## Discussion

Although FBDGs have been implemented across multiple countries, data surrounding their influence and success rate is limited. One literature review assessing FBDGs across four diverse countries found that nutritional surveys were not sufficient to determine the impact of FBDGs on dietary habits within a population [27]. As well as the overall lack of published research on the evaluation of FBDGs, many factors impede the success of FBDGs across various populations. Although some countries with FBDGs have an official body responsible for the implementation of these guidelines, some lacked a plan, strategy, or budget for guideline implementation. Further, in countries where FBDGs have been implemented for several years, there is a noticeable lack of monitoring or evaluation data [5].

This study revealed that approximately 15% of the participants recognize the Healthy Food Palm and the Saudi Healthy Plate guidelines by name. This is slightly lower than the results obtained by Tagtow and Raghavan in 2017, which found that 20.2% of 6,464 participants recognized the USDA’s MyPlate guidelines by name [20]. Like this study, a majority of studies [20, 27, 28] in this field use visual images of the FBDGs to ascertain participant familiarity with the guidelines. As Rong et al. [29] note, graphics help “people better understand the semi-quantitative information about each food group and apply dietary guidelines recommendations into practice in their daily lives.”[29], p. 220].Thus, focusing on image recognition, in this study 15.9% and 30.3% of respondents indicated that they had previously seen the Healthy Food Palm and Saudi Healthy Plate images, respectively.

The Saudi Healthy Plate guidelines were established in Saudi Arabia in 2020, while the Healthy Food Palm guidelines were established back in 2012. It is plausible that the more recent release of the Saudi Healthy Plate guidelines is a key factor in explaining why almost twice as many participants recognized images from the Saudi Health Plate as compared to the Healthy Food Palm image. However, in a study of dietary guidelines in Arabic-speaking countries, Coats et al. [30], found that “Graphical representation of culturally appropriate food choices and servings sizes,” which the Saudi Healthy Palm uses, “is a useful education tool for clinicians and registered dietitians” [30], p. 1130].

In contrast to these findings, a 2018 study of the USDA MyPlate guidelines found that almost two-thirds of 23,343 American participants were able to recognize the MyPlate icon [31]. This number is significantly higher than the findings established here and may be worth investigation and inquiry into the factors that contributed to a higher guideline recognition rate. It is important to note, however. that recognition of the guidelines, either by name or by image, does not necessarily correlate to knowledge of or an understanding of the guidelines themselves. In a systematic of 1,765 articles, Boylan et al., [32] found that “recognition of [dietary] guidelines did not signify understanding.” [32], p. 1].

In this study, 53% of respondents were able to correctly identify the number of food groups within the Healthy Food Palm guidelines, despite only 15.9% of respondents indicating that they had previously seen the corresponding image. This may indicate that the fundamental message of the guidelines is being heard, regardless of how frequently and in what media the associated image is shared or used. Similarly, 54% of participants gave the correct answer when asked how many food groups were in the Saudi Healthy Plate guidelines.

Approximately half of the respondents were able to correctly identify which foods should be consumed at which levels for both the Healthy Food Palm guidelines and the Saudi Healthy Plate guidelines. Comparatively, a 2013 study in the United States found that 45% of participants recognized the USDA's MyPlate guidelines [33].

Despite the fact that approximately half of the subjects in the current study comprehended the Healthy Food Palm and the Saudi Healthy Plate guidelines, a far lower proportion of respondents reported actually adhering to the guidelines. Among participants who expressed that using the Healthy Food Palm or the Saudi Healthy Plate guidelines was helpful, only approximately 4% self-reported eating five portions of fruits and vegetables a day. This aligns with findings from other countries which have reported low overall adherence to FBDGs [34]. Similarly, a more recent study of adults in the United Kingdom reports low adherence to the government’s Eatwell guidelines [35]. The aforementioned study performed in the United Kingdom indicated that less than 0.1% of the sample adhered to all 9 of the Eatwell guidelines, and approximately 26% of the sample adhered to the fruit and vegetable guideline, which, like the Saudi Healthy Plate guidelines, recommends five portions of fruits and vegetables per day [35].

The findings from the present study demonstrate a preference for the Saudi Healthy Plate guidelines as compared to the Healthy Food Palm. However, approximately 25% of the sample reported that they preferred neither the Healthy Food Palm nor the Saudi Healthy Plate guidelines. This may shed light on the need to include stakeholder perspectives on the concept of dietary guidelines to find “appropriate national dietary advice” [36]. Additionally, further understanding why 53.6% of respondents preferred the Saudi Healthy Plate guidelines, compared to 21.4% who preferred the Healthy Food Palm, could help identify the components of dietary guidelines that the public could more easily comprehend and adhere to. Understanding the popularity of the Saudi Healthy Plate could help to further optimize FBDGs and improve adherence throughout the population. Indeed, much remains to be understood regarding the factors required for successful FBDG creation and implementation.

### Limitations

Data from the present study should be interpreted with caveats in mind. Due to the COVID-19 global pandemic, online recruitment and data collection compliant with social distancing requirements may have introduced some degree of self-reporting bias [37, 38]. The weight and height of participants were not objectively measured, which could have resulted in self-reporting bias and/or inaccurate results. Due to the nature of self-reported data, participants may not have answered all questions truthfully or may have rated themselves higher due to social desirability response bias [39]. Therefore, the study findings are affected and limited by these biases.

Further, participants consisted of a non-probability convenience sample that relied on a snowball sampling technique. Unfortunately, this method does not provide an equal participation opportunity to the wider Saudi population. As well,, participants were invited to the study via popular social media platforms. Therefore, findings cannot be generalizable and are not indicative of the Saudi population, particularly those not on social media. Future research might benefit from probability sampling and outreach to participants through multiple channels of recruitment including but not limited to social media platforms. Thus, these findings may not be indicative of the general Saudi population, particularly those not on social media.

Additionally, the study did not collect information on factors such as income, occupation, geographic location, all of which may influence dietary choices and guideline recognition and usage. Further, to more deeply understand and determine the impact of the guidelines on Saudi health – which is confronted by rising numbers of such dietary-related non-communicable diseases as heart disease, diabetes, and obesity – future FBDG research should implement demographic data collection that survey the presence of chronic conditions, including disease, in participants.

Finally, to assess dietary guideline effectiveness in terms of positively influencing health outcomes, future research might benefit from the inclusion of data collection that tracks dietary intake, dietary guideline usage, and health outcomes. In general, although it is possible that FBDGs can contribute to better health outcomes and a reduction in non-transmissible chronic diseases, more research needs to be conducted to better understand dietary choice and behaviors and to more comprehensively understand how to optimize FBDGs. As this study hopes to initiate a dialogue between public health officials, marketing specialists, researchers, and the public, in order to optimize the promotion of dietary guidelines, health officials and researchers should continue to strive to understand the motivating factors behind food consumption and the influence of those factors.

## Conclusions

Overall, findings from the present study indicate that awareness of the current Saudi dietary guidelines is lacking among the general population. Among the participants who were aware of the guidelines, a majority could accurately identify the correct number of food groups and servings recommended by each set of guidelines. However, this did not translate into adherence, with only 10% of respondents indicating that they were following the Saudi Healthy Plate guidelines. Surprisingly, neither social media campaign promotions nor the presence of an influencer was identified by participants as factors that would affect their dietary choices. Rather, participants indicated that food availability and convenience and the state and quality of their own health were the most influencing factors in terms of their dietary choices.

Given the study’s findings, unique opportunities exist for dietary guideline promotion and positive changes in dietary behavior, particularly when a broad spectrum of influencing factors is considered. Future research should not only consider demographic factors but should also look at environmental influences, housing and economic status and stability, and the presence and availability of food, or lack thereof as in a “food desert” [40].

As well, while social media campaigns and influencers may change dietary behaviors for some, making healthy foods more available and convenient, and dietary guideline promotion that shows a stronger nexus between BMI, weight, energy levels, for example, and other markers of personal health, might create more optimal public health engagement.

While the current study offers preliminary insights into the two dominant FBDGs in Saudi Arabia, more research is required to better understand how Saudis view and use the Saudi Healthy Plate and Healthy Palm dietary guidelines. In general, although it is possible that FBDGs can contribute to better health outcomes and a reduction in non-transmissible chronic diseases, more research needs to be conducted to better understand dietary choice and behaviors and to more comprehensively understand how to optimize FBDGs.

## Data Availability

The data that supported the findings of this study are available from the corresponding author on request.

## Abbreviations

BMI: Body mass index
CVD: Cardiovascular diseases
FAO: Food and Agriculture Organization
FBDGs: Food-based dietary guidelines
WHO: World Health Organization

## References

[1] Herforth A, Arimond M, Álvarez-Sánchez C, Coates J, Christianson K, Muehlhoff E (2019). A global review of food-based dietary guidelines. Adv Nutr.

[2] FAO. Food-based dietary guidelines | Food and agriculture organization of the United Nations. 2020. https://www.fao.org/nutrition/education/food-dietary-guidelines/home/en/. Accessed 16 Sep 2022.

[3] Chairs: Joao Breda, Christopher Birt. 7. L. Regular workshop: Public Health Nutrition: Major Policy Areas in Need of Decisions: Organised by: WHO Regional Office for Europe/EUPHA Section on Food and Nutrition. Eur J Public Health. 2015;25(suppl_3):ckv173-055.

[4] World Health Organization (1998). Preparation and use of food-based dietary guidelines: report of a joint FAO/WHO consultation. World Health Organ Tech Rep Ser..

[5] Wijesinha-Bettoni R, Khosravi A, Ramos AI, Sherman J, Hernandez-Garbanzo Y, Molina V (2021). A snapshot of food-based dietary guidelines implementation in selected countries. Glob Food Sec.

[6] MOH News - Dr. Al-Rabeeah Inaugurates the the Dietary Guidelines for Saudis “Healthy Food Palm.” https://www.moh.gov.sa/en/Ministry/MediaCenter/News/Pages/News-2013-01-13-001.aspx. Accessed 1 Nov 2021.

[7] Al-Dkheel M. Dietary Guidelines for Saudis. 2012. https://www.moh.gov.sa/en/Ministry/MediaCenter/Publications/Documents/final english الكتاب العلمي إنجليزي.pdf. Accessed 1 Nov 2021.

[8] Davis C, Saltos E (1999). Dietary recommendations and How they have changed over time.

[9] Saudi Food and Drug Authority. The Saudi Healthy Plate. 2020. https://www.sfda.gov.sa/en. Accessed 6 Jun 2021.

[10] The Eatwell Guide - NHS. https://www.nhs.uk/live-well/eat-well/food-guidelines-and-food-labels/the-eatwell-guide/. Accessed 4 Jun 2022.

[11] Halawani R, et al. Saudi Population's Adherence to the Healthy Food Palm: A Cross-sectional Study. The FASEB Journal. 2019;33(S1):755.4.

[12] Alkhaldy AA, Alamri RS, Magadmi RK, Elshini NY, Hussein RAEH, Alghalayini KW (2019). Dietary adherence of Saudi males to the Saudi dietary guidelines and its relation to cardiovascular diseases: a preliminary cross-sectional study. J Cardiovasc Dev Dis.

[13] Aldubayan K, Aljuraiban G, Aldisi D (2019). Necessary knowledge and skills for dietitians in Saudi Arabia: a qualitative study. Malays J Med Sci.

[14] Hakim N, Alsini N, Kutbi H, Mosli R, Eid N, Mulla U (2020). Knowledge status of dietary guidelines and portion sizes in Saudi Arabian mothers; a cross-sectional study. J Food Nutr Res.

[15] CITC. Saudi Internet report. 2020. https://www.cst.gov.sa/en/indicators/saudiinternet/internt-saudi-2021.pdf. Accessed 2 Jun 2023.

[16] The Global Statistics. Saudi Arabia social media statistics 2022 | Most popular platforms. The Global Statistics. 2022. https://www.theglobalstatistics.com/saudi-arabia-social-media-users/. Accessed 7 Jun 2022.

[17] Eysenbach G. Improving the quality of web surveys: The checklist for reporting results of internet E-surveys (CHERRIES). J Med Internet Res 2004;6(3):e34 https://www.jmir.org/2004/3/e34. 2004;6:e132. 10.2196/jmir.6.3.e34 PMC1550605 15471760

[18] Vandenbroucke JP, von Elm E, Altman DG, Gøtzsche PC, Mulrow CD, Pocock SJ (2014). Strengthening the reporting of observational studies in epidemiology (STROBE): explanation and elaboration. Int J Surg.

[19] Boynton PM, Greenhalgh T (2004). Selecting, designing, and developing your questionnaire. BMJ.

[20] Tagtow A, Raghavan R (2017). Assessing the reach of MyPlate using national health and nutrition examination survey data. J Acad Nutr Diet.

[21] Schwartz JL, Vernarelli JA (2019). Assessing the public’s comprehension of dietary guidelines: use of MyPyramid or MyPlate is associated with healthier diets among US adults. J Acad Nutr Diet.

[22] Sample size calculator by Raosoft, Inc. http://www.raosoft.com/samplesize.html. Accessed 26 Jan 2022.

[23] Population Estimates. The General Authority for statistics. 2020. https://www.stats.gov.sa/en/43. Accessed 3 Feb 2021.

[24] Funkhouser E, Vellala K, Baltuck C, Cacciato R, Durand E, McEdward D (2017). Survey methods to optimize response rate in the national dental practice-based research network. Eval Health Prof.

[25] Ballal R. Attitude and knowledge of self-medication with antibiotics among the public in Riyadh, Saudi Arabia. Asian J Pharma (AJP). 2019;13(02).

[26] Alqahtani A, Aldahish A, Krishnaraju V, Alqarni M, Hassan MAS (2021). General public knowledge of coronavirus disease 2019 (Covid-19) at early stages of the pandemic: a random online survey in saudi arabia. Patient Prefer Adher.

[27] Keller I, Lang T (2008). Food-based dietary guidelines and implementation: lessons from four countries–Chile, Germany. N Z South Afr J Public Health Nutr.

[28] Painter J, Rah JH, Lee YK (2002). Comparison of international food guide pictorial representations. J Am Diet Assoc.

[29] Rong S, Liao Y, Zhou J, Yang W, Yang Y (2021). Comparison of dietary guidelines among 96 countries worldwide. Trends Food Sci Technol.

[30] Coats L, Bernstein J, Dodge E, Bechard L, Aboul-Enein BH (2019). Food-based dietary guidelines of Arabic-speaking countries: a culturally congruent profile. Public Health Nutr.

[31] Jahns L, Conrad Z, Johnson LK, Raatz SK, Kranz S (2018). Recognition of federal dietary guidance icons is associated with greater diet quality. J Acad Nutr.

[32] Boylan S, Louie JCY, Gill TP (2012). Consumer response to healthy eating, physical activity and weight-related recommendations: a systematic review. Obes Rev.

[33] Uruakpa F, Moeckly B, Fulford L, Hollister M, Kim S (2013). Awareness and use of MyPlate guidelines in making food choices. Procedia Food Sci..

[34] Leme ACB, Hou S, Fisberg RM, Fisberg M, Haines J (2021). Adherence to food-based dietary guidelines: a systemic review of high-income and low-and middle-income countries. J Nutrients.

[35] Scheelbeek P, Green R, Papier K (2020). Health impacts and environmental footprints of diets that meet the Eatwell Guide recommendations: analyses of multiple UK studies. BMJ Open.

[36] Bergman K, Persson-Osowski C, Eli K, Lövestam E, Elmståhl H, Nowicka P (2018). Stakeholder responses to governmental dietary guidelines: Challenging the status quo, or reinforcing it?. Bri Food J.

[37] Bauhoff S, Michalos AC (2014). Self-report bias in estimating cross-sectional and treatment effects. Encyclopedia of Quality of Life Research.

[38] Gorber SC, Tremblay M, Moher D, Gorber B (2007). A comparison of direct vs. self-report measures for assessing height, weight and body mass index: a systematic review. Obes Rev.

[39] Arnold HJ, Feldman DC (1981). Social desirability response bias in self-report choice Situations. Acad Manag J.

[40] Wilcox S, Sharpe PA, Liese AD, Dunn CG, Hutto B (2018). Socioeconomic factors associated with diet quality and meeting dietary guidelines in disadvantaged neighborhoods in the Southeast United States. Ethn Health.

